# Anti-Diabetic Activities of Jiaotaiwan in db/db Mice by Augmentation of AMPK Protein Activity and Upregulation of GLUT4 Expression

**DOI:** 10.1155/2013/180721

**Published:** 2013-05-29

**Authors:** Na Hu, Lin Yuan, Hui-Jiao Li, Cheng Huang, Quan-Ming Mao, Yong-Yu Zhang, Min Lin, Yin-Qiang Sun, Xiao-Yu Zhong, Peng Tang, Xiong Lu

**Affiliations:** ^1^Experiment Center for Science and Technology, Shanghai University of Traditional Chinese Medicine, Shanghai 201203, China; ^2^School of Pharmacy, Shanghai University of Traditional Chinese Medicine, Shanghai 201203, China; ^3^Centre for Party of Traditional Chinese Medicine Syndrome and Systems Biology, Shanghai University of Traditional Chinese Medicine, Shanghai 201203, China

## Abstract

Jiaotaiwan (JTW), which is composed of *Coptis chinensis* (CC) and cinnamon (CIN), is one of the most well-known traditional Chinese medicines. In this study, we investigated the antidiabetic effects and mechanism of JTW in db/db mice. Results showed that JTW significantly decreased the level of fasting blood glucose and improved glucose and insulin tolerance better than CC or CIN alone. JTW also effectively protected the pancreatic islet shape, augmented the activation of AMP-activated protein kinase (AMPK) in the liver, and increased the expression of glucose transporter 4 (GLUT4) protein in skeletal muscle and white fat. AMPK and GLUT4 contributed to glucose metabolism regulation and had an essential function in the development of diabetes mellitus (DM). Therefore, the mechanisms of JTW may be related to suppressing gluconeogenesis by activating AMPK in the liver and affecting glucose uptake in surrounding tissues through the upregulation of GLUT4 protein expression. These findings provided a new insight into the antidiabetic clinical applications of JTW and demonstrated the potential of JTW as a new drug candidate for DM treatment.

## 1. Introduction

Diabetes mellitus (DM) is a chronic metabolic disorder characterized by deregulation of glucose and lipid metabolism [1]. With the development of the social economy, DM, especially type 2 diabetes mellitus (T2DM), has become a serious public health problem [2]. Numerous drugs, such as rosiglitazone (ROS) and metformin (MET), have been used in the treatment of DM. However, treatment with synthetic drugs has been reported to lead to various side effects [3]. Therefore, searching for better agents from herbs or natural products that can be used to treat diabetes is necessary [4]. 

Traditional Chinese medicines (TCMs), which have been used by the Chinese to treat illnesses for thousands of years, are combination drugs comprising several different active compounds. TCMs are better at controlling complex disease systems such as diabetes and are less prone to causing drug resistance development [5]. Jiaotaiwan (JTW) is one of the most well-known TCMs from *Han's Book on Medicine* compiled by Mao Han. JTW is composed of *Coptis chinensis* (CC) and cinnamon (CIN). In China, JTW is mainly used to treat insomnia. The major active constituents of CC and CIN reduce blood glucose levels [6–8]. Berberine chloride, which is isolated from CC, possesses anti-diabetic activity [9, 10]. Cinnamaldehyde is a major active constituent isolated from CIN. Studies show that cinnamaldehyde can reduce the fasting blood glucose level in rats treated with streptozotocin [11]. The therapeutic effect of JTW is also reportedly better than that of CC or CIN alone for treating type 2 diabetes mellitus (DM) in rats [12]. However, the mechanism of the glucose-decreasing effect of JTW is equivocal.

Hepatic glucose production and glucose uptake in surrounding tissues are the key in glucose homeostasis. AMP-activated protein kinase (AMPK) has a critical function in this process because it can suppress gluconeogenesis in the liver and promote glucose uptake in peripheral tissues [13]. The glucose uptake in surrounding tissues is mediated by glucose transporter4 (GLUT4) [14]. Research shows that as a result of the up-regulation of GLUT4 protein expression in adipose cell and skeletal muscle in a state of insulin resistance, glucose uptake in the adipose cell and skeletal muscle is promoted, and glucose tolerance and insulin resistance are improved [15, 16].

In this study, we investigated the anti-diabetic effects of JTW in typical T2DM model db/db mice. We detected the AMPK protein levels in the liver and the GLUT4 protein levels in skeletal muscle and white fat to validate the beneficial effects of JTW as an anti-diabetic agent and to clarify the mechanisms of its action.

## 2. Materials and Methods

### 2.1. Animals

The animal protocols used in this study were approved by the Shanghai University of Traditional Chinese Medicine for Animal Studies (Approval number 10032). Female db/m and db/db mice (C57BL BKS cg-M+/+ lepr−/−) purchased from the SLAC Laboratory (Shanghai, China) were housed at 22 ± 2°C and 55% ± 5% relative humidity, with a dark cycle of 12 h (19:00 to 07:00) and a light cycle of 12 h (07:00 to 19:00). Eight-week-old db/db mice were used in the experiment. The db/db mice were randomly divided into five groups, namely, model control group (Model), JTW-treated group (JTW), CC-treated group (CC), CIN-treated group (CIN), and ROS-treated group (ROS). The db/m mice not included in the five groups were designated as the normal control group (Normal). Each group comprised eight mice. JTW (8.4 g/kg), CC (7.6 g/kg), CIN (0.76 g/kg), ROS (5 mg/kg), or water was intragastrically administered to the mice for four weeks. The model control (Model) and normal control (Normal) groups were treated with water.

### 2.2. Preparation of JTW, CC, and CIN

Composition proportions of JTW prescription: the composition of CC : CIN was equal to 10 : 1. The drugs were purchased from the Yanghetang Decoction Pieces Limited Company (Shanghai, China) and extracted by the Analyses and Testing Laboratory in Shanghai University of Chinese Medicine. The extraction steps of JTW were performed as follows: CIN was soaked with 6 volumes of water for 2 h. The volatiles (A), drug liquid (B), and drug residue (C) were obtained with a simultaneous distillation and extraction (SED) equipment for 5 h. D (CC, B, and C) was soaked with 5 volumes of water for 0.5 h and then subsequently boiled for 1 h and extracted twice to obtain the extraction solution (E). Drug concentrations (F) were obtained from E using low heat. A and F were mixed to form JTW with a concentration of 0.84 g/mL. 

The extraction steps of CC concentrations were indicated as follows: CC was soaked with 5 volumes of water for 0.5 h and then subsequently boiled for 1 h and extracted twice to obtain the extraction solution. Subsequently, the CC concentrations were obtained from the extraction solution using low heat. 

The extraction steps of CIN concentrations were indicated as follows: CIN was soaked with 6 volumes of water for 2 h. The volatiles (A), drug liquid (B), and drug residue (C) were obtained with an SED equipment for 5 h. A and B were mixed to form CIN concentrations. The concentrations of CC and CIN were 0.76 and 0.076 g/mL, respectively.

### 2.3. Intraperitoneal Glucose Tolerance Test and Intraperitoneal Insulin Tolerance Test

After two and three weeks of treatment, db/db mice were fasted overnight (12 h). Glucose levels were determined from the tail vein (0 min) before the injection of glucose (1 g/kg body weight) or insulin (1 u/kg body weight). Additional blood samples were collected at regular intervals (15, 30, 60, and 120 min) for glucose measurement.

### 2.4. Histology

All mice were sacrificed after four weeks of treatment, and their pancreas were immediately dissected. All of the mice pancreases were fixed in 10% neutral formalin, desiccated, and then embedded in paraffin. The pancreases were sectioned (3 *μ*m thick), and the sections were transferred to gelatin-coated slides. Then, the slides were stained with hematoxylin and eosin (HE) and examined under light microscope. 

### 2.5. Immunohistochemistry

The sections were deparaffinized with xylene, rehydrated in graded ethanol (100% to 95%), and rinsed with water and 0.1 M phosphate-buffered saline (PBS, pH 7.4). Following 10 min treatment with 3% H_2_O_2_ at room temperature, the sections were washed with 0.1 M PBS. The sections were incubated with an anti-insulin monoclonal antibody (Boster, China; diluted 1 : 200) at 37°C for 1 h. After they were washed with PBS, the sections were allowed to react with goat anti-rabbit IgG with horse radish peroxidase (HRP) (MiaoTong, China, diluted 1 : 500) at room temperature for 20 min and then washed with PBS. HRP activity was developed by DAB. Sections were then counterstained with hematoxylin and examined under a light microscope.

### 2.6. Transmission Electron Microscopy

The mice pancreases (1 mm^3^) were fixed in 2% glutaraldehyde in 0.1 M D-PBS for 2 h at 4°C. Following three washes with 0.1 M PBS was postfixed in 1% osmium tetroxide for 2 h and dehydration in ascending concentrations of ethanol and acetone (30% to 50% ethanol, 70% ethanol-uranyl acetate, 80% ethanol, 100% ethanol-acetone, and 100% acetone). Subsequently the pancreases were embedded in Epon 618. Thin 80 nm sections were prepared on a LEICA ULTRACUT R ultramicrotome and stained with lead citrate. The sections were examined with a transmission electron microscope.

### 2.7. Western Blot Analysis

Equal amounts of protein from each sample, 120 *μ*g from hepatic tissue and skeletal muscle, and 240 *μ*g from white fat were separated on SDS-PAGE gels and then transferred to PVDF membranes. Blots were blocked with 5% nonfat milk in Tris-buffered saline with 0.1% Tween-20 (TBST, pH 8.0 25 mM Tris, 137 mM NaCl, 2.7 mM KCl, and 0.1% Tween-20) at room temperature for 1 h, followed by overnight incubation with primary antibodies at 4°C. The blots were hybridized with secondary antibody-conjugated HRP in 5% nonfat milk dissolved in TBST at room temperature for 2 h after they were washed with TBST three times. Protein expression was visualized using the ECL Western Blotting Detection System after three washes with TBST.

### 2.8. Statistical Analysis

The results were expressed as mean ± standard error of mean (*x* ± SE). Data analyses were performed using SPSS15.0 software. *t*-test and one-way ANOVA were adopted for general data analysis. LSD method was applied for comparisons between groups. Data were considered statistically significant when *P* < 0.05.

## 3. Results

### 3.1. HPLC Profiles of JTW

**Figure 1:**
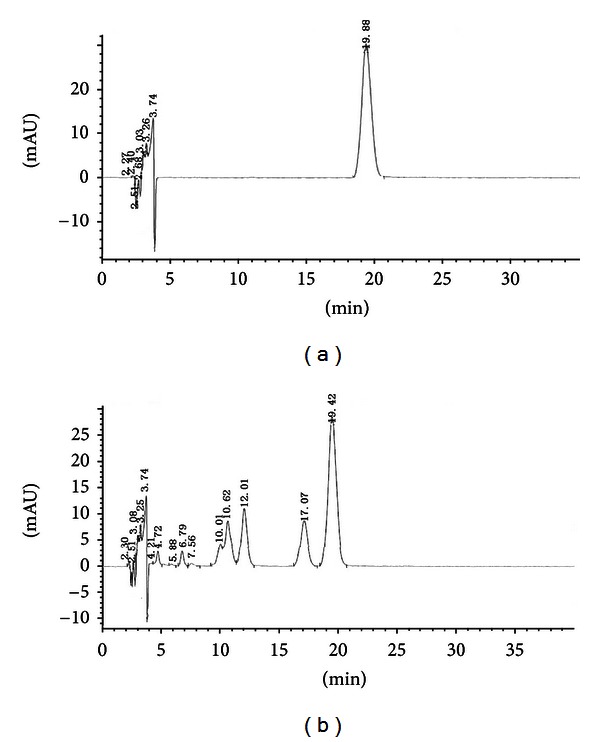
HPLC profiles of JTW and reference material of berberine hydrochloride. The chromatographic conditions were indicated as follows: chromatographic column, C18 (4.6 × 250 mm^2^, 5 *μ*m); flow rate, 1 mL/min; mobile phase, 0.05 M methyl cyanide and potassium dihydrogen phosphate (25 : 75); column temperature, 30°C; examination wave length, 346 nm; and sample volume, 20 *μ*L. (a) The peak area of the reference material of berberine hydrochloride was 1481.31 and the retention time was 19.39 min. (b) The peak area of JTW was 1402.66 and the retention time was 19.41 min. Given the formula (*A*_1_/*C*_1_ = *A*_2_/*C*_2_), the berberine concentration in the three JTW samples was 25.61 mg/mL (*A*_1_ = 1481.31, *C*_1_ = 0.02, and *A*_2_ = 1402.66).

To investigate the stability of the JTW water decoction, we repeated the same extraction step times to obtain JTW and then determined the berberine hydrochloride content in JTW by HPLC analysis. The berberine concentration in the JTW samples was 25.61 mg/mL based on the formula, which indicated that the berberine hydrochloride content of JTW was stable (Figure 1).

### 3.2. Effects of JTW on Body Weight, Food Intake, and Water Intake in db/db Mice

**Figure 2:**
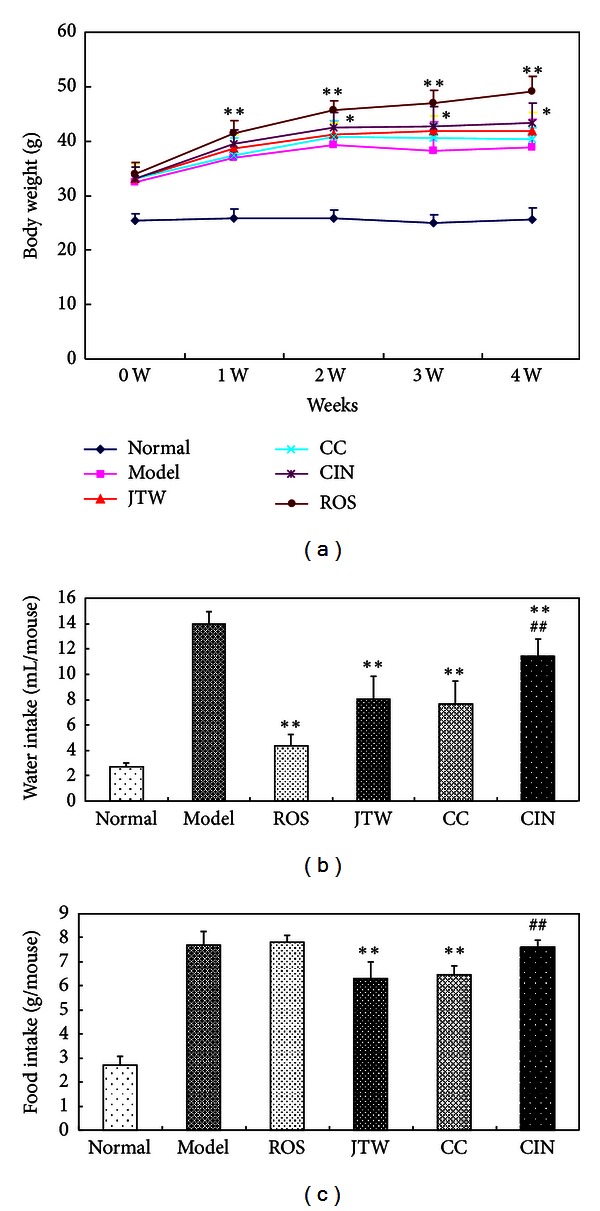
Effects of JTW on body weight, water intake, and food intake in db/db mice. (a) Body weight: body weight was measured every week after treatment (*n* = 8). (b) Water intake: mice were administered with JTW each day for two weeks (8.4 g kg^−1^ day^−1^), CC (7.6 g kg^−1^ day^−1^), CIN (0.76 g kg^−1^ day^−1^), or ROS (5 mg kg^−1^ day^−1^) in a vehicle using oral gavage. The water intake amount was recorded every 24 h throughout the treatment (*n* = 8). (c) Food intake: after treatment with gastric infusion for two weeks, water intake was recorded every 24 h throughout the treatment (*n* = 8). The data were shown as mean ± SE. *P < 0.05 compared with the model control group; **P < 0.01 compared with the model control group; ^##^ *P* < 0.01 compared with the JTW-treated group.

No significant weight gain was observed in the JTW and CC groups after four weeks of treatment. By contrast, the mice began to gain weight after two weeks of treatment in the CIN group and after one week of treatment in the ROS group (Figure 2(a)). All of the drugs used reduced the level of water intake in db/db mice, but only JTW and CC decreased the level of food intake (Figures 2(b) and 2(c)).

### 3.3. JTW Ameliorates Glucose Tolerance and Insulin Tolerance in db/db Mice

**Figure 3:**
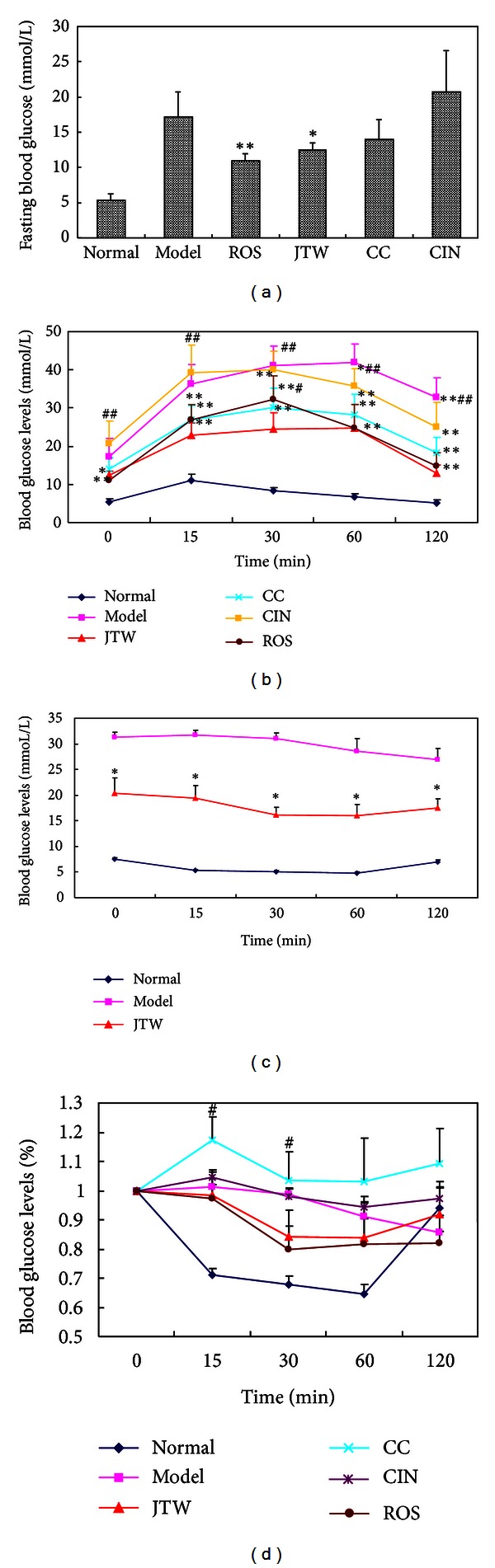
Effects of JTW on fasting blood glucose, glucose tolerance, insulin tolerance, and fasting serum insulin level in db/db mice. (a) Fasting blood glucose levels after two-week treatment (*n* = 8). (b) Intraperitoneal glucose tolerance test (IPGTT) after two-week treatment. The mice were fasted for 12 h before measuring blood glucose levels at 0 min. A total of 1 g/kg body weight of glucose was intraperitoneally injected, and glucose levels were tested at regular intervals of 15, 30, 60, and 90 min (*n* = 8). (c), (d) Intraperitoneal insulin tolerance test (IPITT) was performed after three-week treatment. Glucose levels were tested in the same way after intraperitoneally injecting 1 *μ*/kg body weight of insulin (*n* = 8). Data are presented as mean ± SE. *P < 0.05, **P < 0.01 versus the model control group. ^#^ *P* < 0.05, ^##^ *P* < 0.01 versus JTW-treated group.

To understand the effects of JTW on blood glucose of db/db mice, fasting blood sugar levels were examined and shown in Figure 3(a). Blood glucose levels in JTW-treated db/db mice were significantly lowered. However, the levels were not significantly changed in the CC- and CIN-treated groups. T2DM commonly coexists with impaired glucose tolerance and insulin tolerance [17]. To verify whether JTW improved glucose tolerance and insulin tolerance *in vivo*, we measured glucose tolerance, insulin tolerance in JTW-treated db/db mice.  Figure 3(b) showed that glucose tolerance in JTW mice improved at 0, 15, 30, 60, and 120 min, and CC and CIN improved glucose tolerance at 15, 30, 60, 120 min, 60 and 120 min, respectively. Upon comparison of the CC- and CIN-treated groups with the JTW-treated group, we observed that glucose tolerance in JTW mice was better than that in the CC group at 30 min and that in the CIN group at all time periods, indicating that JTW could ameliorate glucose tolerance and exhibit better performance than CC and CIN.  Figure 3(c) showed that the insulin tolerance in JTW mice improved at 0, 15, 30, 60, and 120 min compared with that in the model group.  Figure 3(d) also indicated that the downward shift of blood glucose levels in the JTW group mice after treatment with insulin was more noticeable at 15 and 30 min than in the CIN group. 

### 3.4. JTW Ameliorates Islets Morphology and Function

**Figure 4:**
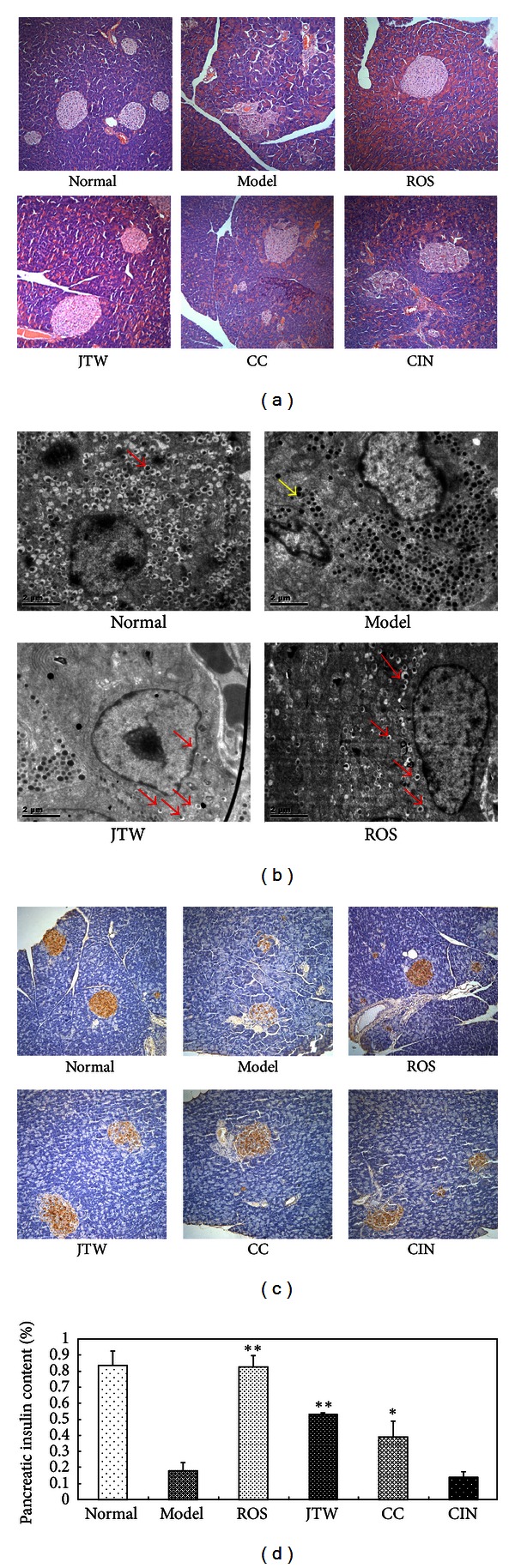
JTW ameliorates islet morphology in pancreas and *β*-cell function. (a) HE stain of pancreas sections, 200*x* (*n* = 8). (b) Images of pancreatic islet *β* cells under TEM, 6000x. The samples were prepared as described in Section 2, and pictures of the cells in the islet center were taken to observe *β* cell morphology by TEM; (black arrow)*β*-SGs, (white arrow)*α*-SGs (*n* = 8). (c) Immunohistochemical stain of insulin in pancreas sections, 200x (*n* = 8). (d) Pancreatic insulin content. After insulin immunohistochemical staining, the deeply stained insulin-positive cells in pancreatic islets were tested with an I-solution Image Analysis System (*n* = 8). Data are presented as the mean ± SE. *P < 0.05, **P < 0.01 versus the model control group.

To understand the effects of JTW on islet morphology and function in db/db mice, HE staining, transmission electron microscopy (TEM), and immunohistochemistry analyses were performed. HE stain results showed that the islets in the JTW group had a relatively regular shape and reduced infiltration of exocrine glands. A larger number of islet cells and occasional vacuolar degeneration than the model group were also observed (Figure 4(a)). 

We also observed *β*-cell morphology by TEM. Damage to the pancreatic islet *β*-cell structure and function results from insulin resistance [18]. In normal group, TEM generally showed numerous *β* cells in the islet center, and these cells secreted many global granules (*β*-SGs) (Figure 4(b) (black arrow)). Few *α* cells also existed in the islet periphery, and these cells secreted *α* secretory granules (*α*-SGs). The space between the membrane and core of *β*-SGs was large, clear, and bright. The electron density of *α*-SGs was higher than that of *β*-SGs, and the space between the membrane and core was small. 

The results showed numerous *α*-SGs in the islet center in the model group (Figure 4(b)(white arrow)), which indicated that the *β* cells were disabled and occupied by *α* cells. However, some residual *β*-SGs were observed in the *β* cells of the JTW group (Figure 4(b) (black arrow)), which indicated that JTW may exert a protective effect on the beta cells. Insulin immunohistochemistry was used to determine the pancreatic insulin content, and the I-solution Image Analysis System was used to test the deeply stained insulin-positive cells in the pancreatic islets. The results showed that the pancreatic insulin content of the JTW group increased compared with the model group but not compared with the ROS group (Figures 4(c) and 4(d)). This finding indicated that the main mechanism of the glucose-decreasing effect of JTW may differ from that of ROS.

### 3.5. JTW Induced AMPK in the Liver and Increased the Expression of GLUT4 in Skeletal Muscle and White Fat in db/db Mice

**Figure 5:**
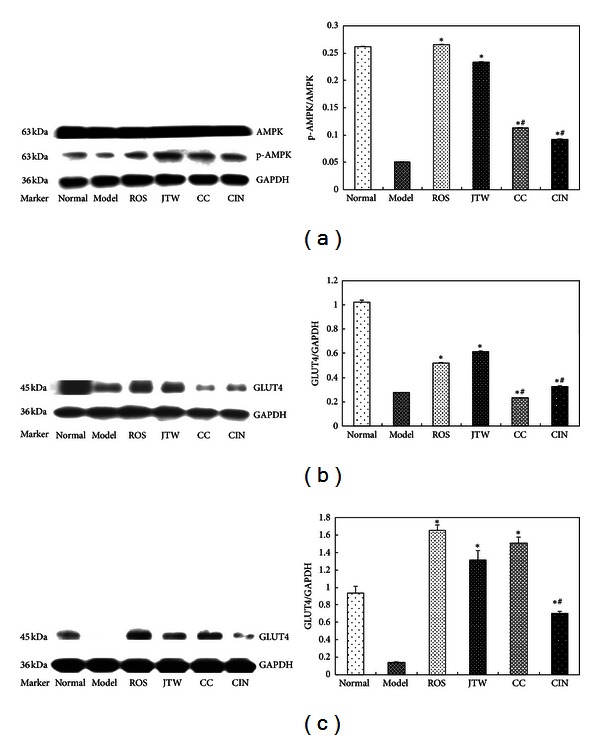
JTW induced AMPK in the liver and increased the expression of GLUT4 protein in skeletal muscle and white fat in db/db mice. (a) Expression of AMPK protein and p-AMPK protein in the liver (*n* = 3). (b) Expression of GLUT4 protein in abdominal fat (*n* = 3). (c) Expression of GLUT4 protein in skeletal muscle (*n* = 3). Data are presented as mean ± SE for twelve-week-old mice per group. *P < 0.05 versus model control group. ^#^ *P* < 0.05 versus JTW-treated group.

AMPK activation and GLUT4 expression are crucial for the treatment of diabetes [19, 20]. To evaluate whether JTW has a positive effect on AMPK and GLUT4 protein, hepatic tissue, skeletal muscle, and abdominal fat were dislodged, and then phosphorylation of AMPK in the liver and expression of GLUT4 in muscle or fat were determined by Western blot analysis with anti-pAMPK-Thr172 antibody and anti-GLUT4 antibody. As expected, the results showed that JTW induced phosphorylation of AMPK in hepatic tissues, and its performance was more effective than that of single herbs (Figure 5(a)). This finding indicates that JTW can effectively contribute in the inhibition of gluconeogenesis and is regulated by AMPK. The glucose uptake in the adipose cell and skeletal muscle is mediated by GLUT4. The experimental findings proved that the expression of GLUT4 significantly increased in both skeletal muscle and abdominal fat of db/db mice compared with those of the model control group (Figures 5(b) and 5(c)), revealing that JTW enhanced glucose uptake in surrounding tissues by upregulating the protein expression of GLUT4.

## 4. Discussion

JTW is one of the most well-known TCMs formulae; it is composed of CC and CIN. In this study, we investigated the hypoglycemic action of JTW, and the results confirmed that JTW possessed anti-diabetic activities *in vivo*. To determine whether the activity of JTW was formed by the overprint of CC and CIN, db/db mice were administered equal volumes of CC and CIN and designated as the control group. The results showed that the administration of single herb did not lower the fasting blood glucose levels in db/db mice after two weeks of treatment. The findings of this study differed from those of other researchers who found that CC and CIN had anti-diabetic properties [6–8]. This result may be due to the disparity in experimental subjects, dosages, and methods for testing. The dosage of JTW was predetermined in another experiment. Two JTW doses were designed for the treatment of mice. One dose was 2.1 g/kg (JTW1) and the other was 8.4 g/kg (JTW2). Results showed that JTW (8.4 g/kg) significantly decreased the level of fasting blood glucose (see Supplementary Material a available online at http://dx.doi.org/10.1155/2013/180721) and improved glucose tolerance (see Supplementary Material b), although both doses of JTW reduced the level of water intake (see Supplementary Material c), food intake (see Supplementary Material d), and urine volume (see Supplementary Material e) in db/db mice. Therefore, we used 8.4 g/kg in subsequent experiments. We decided on the dosage of CC and CIN based on the fact that 8.4 g/kg JTW was composed of 7.6 g/kg CC and 0.76 g/kg CIN. In our study, we found that water intake, food intake, and fasting blood glucose levels significantly decreased in the JTW-treated group. CC and CIN did not improve the common symptoms of diabetes in db/db mice, although water and food intake decreased in the CC group. Conversely, water intake, food intake, and body weight increased in the CIN group. This result revealed that the compatibility of CC and CIN had scientific significance. 

T2DM typically coexists with impaired glucose tolerance and insulin tolerance. Thus, we measured glucose tolerance and insulin tolerance in JTW-treated db/db mice. The result showed that glucose tolerance in JTW mice significantly improved at 0, 15, 30, 60, and 120 min and were better than those of CC and CIN groups, confirming that JTW could ameliorate glucose tolerance and have more powerful action than CC and CIN. This result agreed with the insulin tolerance results. Our findings suggest that the compatibility of CC and CIN has scientific significance, which may be associated with the bioavailability enhancement of the main component as a result of interaction with each other, indicating that this topic is worthy of further study.

Protection of *β* cells is significant in T2DM treatment [21, 22]. To understand the effects of JTW on the pancreas shape in db/db mice, HE staining, TEM, and immunohistochemistry test were performed to observe the histomorphology of the pancreas. The results showed that pancreatic islet shape and *β*-cell function in the JTW group improved to a certain extent, but the effect of this improvement was not really as good as that of ROS, especially in the result of pancreatic insulin content. Therefore, we hypothesized that JTW has other hypoglycemic mechanisms. ROS belongs to a class of drugs known as peroxisome proliferator-activated receptor (PPAR) agonists. PPAR*γ* has major functions in regulating glucose homeostasis and lipogenesis [23, 24]. Studies have proved that PPAR*γ* agonists are potent insulin-sensitizing agents for treating T2DM but can induce body weight gain in patients [25, 26]. The conclusions were in agreement with the ROS results in this study. Significantly, our results showed that JTW did not increase body weight upon blood sugar reduction in db/db mice, revealing that JTW blocked the side effect of weight gain. Therefore, we posited that the hypoglycemic mechanism of JTW may not be directly connected to PPAR, and the hypoglycemic mechanism of JTW from other signaling pathways should be studied. 

 Hepatic glucose production and glucose uptake in surrounding tissues are highly important in body glucose homeostasis. We assumed that JTW is effective in inhibiting hepatic gluconeogenesis and promoting glucose uptake of peripheral tissue. Increased hepatic glucose production is a major cause of hyperglycemia in T2DM. Gluconeogenesis and glycogenolysis are two methods of hepatic glucose production [27], but gluconeogenesis is more important. AMPK is an *αβγ* heterotrimer that has a key function in regulating glucose homeostasis and lipogenesis, comprising an *α*-catalytic subunit with *βγ*-regulatory subunits. AMPK is important in regulating gluconeogenesis [13]. AMPK phosphorylation can directly phosphorylate CREB-regulated transcription coactivator 2 on Ser171, which would be antagonistic to the induction of gluconeogenic genes [28]. On the other hand, glucose uptake in surrounding tissues is mediated by GLUT4 [14]. GLUT4 is the major glucose transporter of muscle and adipose tissues and facilitates glucose delivery to intracellular from extracellular, thus augmenting glucose uptake. GLUT4 mRNA and protein content can decrease in peripheral tissues, which may be one of the reasons for insulin resistance [29]. To evaluate the effect of JTW on AMPK protein in the hepatic tissue and GLUT4 protein in the peripheral tissue, Western blot analysis was used in our study. As expected, the data showed that JTW significantly induced AMPK phosphorylation in hepatic tissues and increased the expression of GLUT4 protein in both skeletal muscle and abdominal fat of db/db mice compared with the control group. The results showed that JTW increased the expression of GLUT4 that may reflect functional GLUT4 located in cellular surface, but this result requires further study. Anyway, these results indicated that JTW effectively inhibited AMPK-regulated gluconeogenesis and enhanced glucose uptake in surrounding tissues by upregulating the expression of GLUT4. The effects of JTW on downstream protein and AMPK gene require further studies. 

## 5. Conclusion

We prove in this study that JTW reduces the blood glucose levels, food intake, and water intake and ameliorates glucose tolerance and insulin tolerance in db/db mice. As a result of its safety and low cost, especially its fewer side effects, JTW has high potential in regulating glucose metabolism. Our data prove that JTW has multiple targets in the hypoglycemic mechanism, such as suppressing gluconeogenesis through AMPK activation in the liver and affecting glucose uptake of surrounding tissues by upregulating the protein expression of GLUT4. These findings suggest that JTW may be used as a potential candidate for T2DM therapy.

## Supplementary Material

The experiment of JTW doses: Eight-week-old db/db mice were used in the experiment and randomly divided into four groups, namely, model control group (Model), JTW1 treated group (JTW1), JTW2 treated group (JTW2), and ROS-treated group (ROS). The db/m mice were designated as the normal control group (Normal). Each group comprised eight mice. JTW1 (2.1 g/kg), JTW2 (8.4 g/kg), ROS (5mg/kg), or water was intragastrically administered to the mice for two weeks. The model control (Model) and normal control (Normal) groups were treated with water. Effects of JTW1 and JTW2 on the level of fasting blood glucose, glucose tolerance, water intake, food intake and urine volume in db/db mice were indicated as follows.
