# Supplementary material for: Anti-Diabetic Activities of Jiaotaiwan in db/db Mice by Augmentation of AMPK Protein Activity and Upregulation of GLUT4 Expression
Source: Evid Based Complement Alternat Med. 2013 May 29;2013:180721. doi: 10.1155/2013/180721 (PMC3681272; doi:10.1155/2013/180721)
Supplement: Supplementary file 1 — The experiment of JTW doses: Eight-week-old db/db mice were used in the experiment and randomly divided into four groups, namely, model control group (Model), JTW1 treated group (JTW1), JTW2 treated group (JTW2), and ROS-treated group (ROS). The db/m mice were designated as the normal control group (Normal). Each group comprised eight mice. JTW1 (2.1 g/kg), JTW2 (8.4 g/kg), ROS (5mg/kg), or water was intragastrically administered to the mice for two weeks. The model control (Model) and normal control (Normal) groups were treated with water. Effects of JTW1 and JTW2 on the level of fasting blood glucose, glucose tolerance, water intake, food intake and urine volume in db/db mice were indicated as follows. [file 180721.f1.doc]

Supplementary data

a

b

c

d

e

Supplementary data: (a) Fasting blood glucose levels after two weeks treatment. (b) Intraperitoneal glucose tolerance test (IPGTT) after two weeks treatment. The mice were fasted for 12 h before measuring blood glucose levels at 0 min. A total of 0.5 g/kg body weight of glucose was intraperitoneally injected, and glucose levels were tested at regular intervals of 15, 30, 60, and 90 min. (c) Water intake: mice were administered with JTW1, JTW2, ROS each day for two weeks in a vehicle using oral gavage. The water intake amount was recorded every 24 h throughout the treatment. (d) Food intake: After treatment with gastric infusion for two weeks, water intake was recorded every 24 h throughout the treatment. (e) Urine volume: After treatment for two weeks, urine volume was recorded every 6 h throughout the treatment.The data were shown as mean ± SE. Eight-week-old mice were used. N = 8 for all groups. * *P*<0.05 compared with the model control group; ** *P*<0.01 compared with the model control group.
